# Association of perioperative intravenous fluid strategy with acute kidney injury following off-pump coronary artery bypass surgery

**DOI:** 10.1186/s13054-015-1136-x

**Published:** 2015-11-24

**Authors:** Fu-Shan Xue, Gao-Pu Liu, Rui-Ping Li

**Affiliations:** Department of Anesthesiology, Plastic Surgery Hospital, Chinese Academy of Medical Sciences and Peking Union Medical College, 33 Ba-Da-Chu Road, Shi-Jing-Shan District, Beijing, 100144 People’s Republic of China

In the nonrandomized observational study by Kim et al. [1] evaluating association of perioperative intravenous fluid strategy with acute kidney injury (AKI) following off-pump coronary artery bypass surgery, the cumulative amount of intravenous fluids received during surgery and in the immediate postoperative period was significantly different between control and renal protective fluid management groups. We are very interested in knowing whether the serum creatinine (sCr) levels used for diagnosis of postoperative AKI in this study had been corrected based on perioperative fluid balance.

Moore et al. [2] validate that not adjusting sCr levels for fluid balance may underestimate the incidence of AKI and confuse association of AKI with postoperative outcomes in cardiac surgery patients. Furthermore, we were not provided with details of intraoperative hemodynamics instability and drug administration. It has been shown that intraoperative systolic blood pressure decrease relative to baseline is independently associated with AKI after cardiac surgery [3, 4]. Similarly, intraoperative uses of inotropes, vasopressors, and diuretics are also attributable to the development of AKI after cardiac surgery [5].

Finally, increases in sCr and AKI 7 days after surgery are probably subjected to confounding postoperative events, such as low cardiac output syndrome, anemia, hypoalbuminemia, reoperation for bleeding, blood transfusion, sepsis, etc. Also, the postoperative events mentioned above can significantly affect clinical outcomes of patients, for example the extubation time, duration of hospital stay, and mortality. However, it was unclear whether the postoperative events associated with AKI and clinical outcomes were comparable between groups or required statistical adjustments.

We thus argue that addressing these issues would further clarify the transparency of this study.

## Abbreviations

AKI: Acute kidney injury
sCr: Serum creatinine
